# Ecologically expanding the One Health framework to unify the microbiome sciences

**DOI:** 10.1128/mbio.03147-24

**Published:** 2025-05-12

**Authors:** Nichole Ginnan, Sharifa G. Crandall, Madangchanok Imchen, Francisco Dini-Andreote, Tim I. Miyashiro, Vishal Singh, Erika Ganda, Seth R. Bordenstein

**Affiliations:** 1 One Health Microbiome Center, Huck Institutes of the Life Sciences, Pennsylvania State University 124474, University Park, Pennsylvania, USA; 2 Department of Plant Pathology and Environmental Microbiology, The Pennsylvania State University 171650, University Park, Pennsylvania, USA; 3 Department of Biology, The Pennsylvania State University 118135, University Park, Pennsylvania, USA; 4 Department of Plant Science, The Pennsylvania State University 311373, University Park, Pennsylvania, USA; 5 Department of Biochemistry and Molecular Biology, The Pennsylvania State University 189499, University Park, Pennsylvania, USA; 6 Department of Nutritional Sciences, The Pennsylvania State University 200776, University Park, Pennsylvania, USA; 7 Department of Animal Sciences, The Pennsylvania State University 8082, University Park, Pennsylvania, USA; 8 Department of Entomology, The Pennsylvania State University 171648, University Park, Pennsylvania, USA; University of Pretoria, Pretoria, Gauteng, South Africa; Monash University, Melbourne, Victoria, Australia

**Keywords:** microbiome, One Health, symbionts, team science, pathogens, microbes, bacteria, fungi, viruses

## Abstract

The One Health framework, traditionally focused on microbial threats, needs a bold expansion to include the full breadth of microbial diversity—from pathogenic to beneficial—within its ecological and evolutionary context. By shifting focus from disease surveillance to microbial stewardship, an integrative One Health microbiome science approach breaks down traditional silos in microbiome research, accelerating integrative and comparative science to uncover foundational insights into microbial community assembly, stability, and resilience. Ultimately, this will help unlock the full potential of microbiomes to enhance global health and sustainably manage ecosystems.

## PERSPECTIVE

Microbiome research rapidly expanded in the last two decades, with nearly 200,000 peer-reviewed articles that use the word “microbiome” and significant microbial lineage discoveries reshaping the evolutionary tree of life. The unprecedented growth of microbial-focused biotech industries, projected to reach a $317 billion value by 2032 (1), further reflects the increasing recognition that microbial ecology and traits are fundamental drivers of global health. Despite these advancements, the field remains largely compartmentalized within subdisciplines, limiting our ability to develop system-agnostic insights and integrative theories that connect microbial ecology and evolutionary dynamics across systems.

How did the field get here? Microbiome sciences emerged from historically siloed skillsets and scholarship in organismal (e.g., microbiology, botany, and zoology) and organizational (e.g., molecular, cellular, and evolutionary) domains. While the specialized foci launched and propelled research to its current state, there is now an emerging imperative to establish central ideas and standards that incorporate principles of community ecology, evolutionary theory, and systems biology. Once established, this framework will help drive educational pipelines and solidify microbiome science as an accredited discipline within the life sciences at graduate, undergraduate, and high school levels (2).

## AN ECOLOGICALLY EXPANDED ONE HEALTH FRAMEWORK

**Fig 1:**
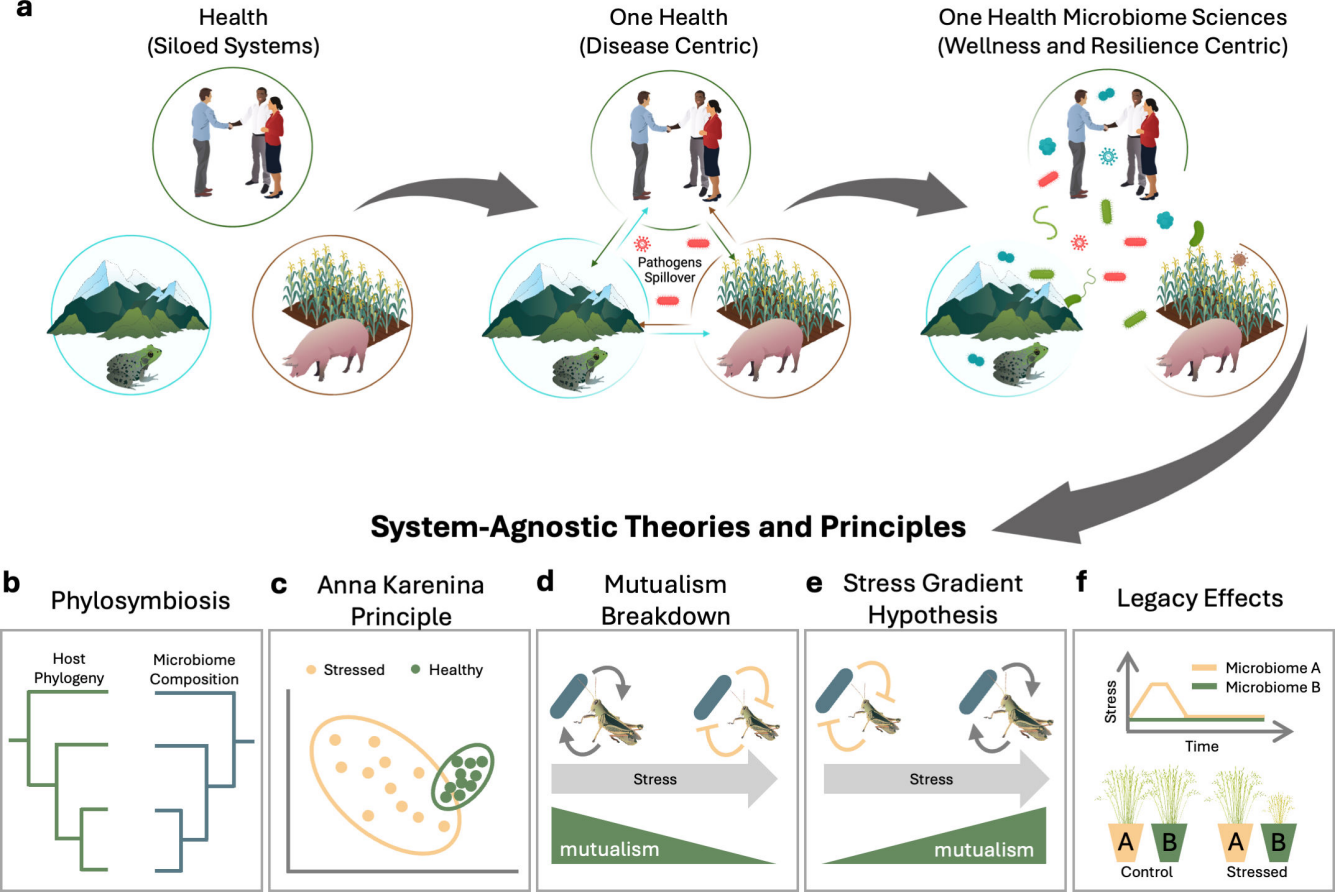
One Health microbiome sciences. (a) An expanded One Health microbiome sciences framework that incorporates the ecology of all microbes, beyond disease-causing pathogens, and grows to focus on ecosystem wellness and resilience. This One Health framework will help unify the microbiome sciences and advance core disciplinary theories and principles with cross-system validation and comparative studies, including a few examples highlighted in this article: (b) phylosymbiosis, host-associated microbiome relationships reflect the host’s evolutionary history; (c) Anna Karenina principle, unhealthy microbiomes fail in many different ways, resulting in more heterogeneity; (d) mutualism breakdown, beneficial relations collapse under stress; (e) stress gradient hypothesis, mutualistic interactions are more common in stressful environments; and (f) legacy effects, past events or conditions impact future community responses and traits.

Inspired by the need to sustainably balance and optimize health across interacting ecosystems, the One Health framework emphasizes the interconnectedness of human, environmental, and agricultural salubrity. One Health studies traditionally focus on microbial threats from agriculture and the environment that can or do impact humans (3), such as pathogen spillover or the spread of antimicrobial resistance (Fig. 1a). One Health also underscores the importance of interdisciplinary collaboration and enhances our understanding of environmental impacts on disease transmission and outcomes, as well as evolutionary pressures driving the emergence of virulence. These attributes are as salient to convergence research in pathogenesis as they are to the microbiome sciences, which seeks to unravel the significance and applications of all microorganisms, whether harmful, helpful, or harmless (4). Therefore, we view the One Health microbiome sciences as an expanding framework to unsilo the historical roots of the field and force a conceptual shift to common rules and trends (Fig. 1a).

For example, large-scale ecological studies, such as the Earth Microbiome Project, mapped global microbial diversity, revealing a significant number of conserved microbial taxa and patterns of environmental filtering and species sorting (5). Other case studies have illuminated how nonpathogenic microorganisms shared between plants and insects prevent fungal pathogen invasions in both systems (6) and how soil-borne microbes promote anti-inflammatory effects in mammals (7). These examples demonstrate the need to explore microbial drivers of health and their role in ecosystem stabilizing feedbacks. Expanding One Health to encompass the vast spectrum of microbial diversity, effects, and environmental range will help shift the focus from disease surveillance to microbial stewardship, sustainability, health maintenance, and climate change resilience across interconnected systems. Furthermore, this framework emphasizes comparative studies and interdisciplinary collaborations, creating a robust foundation for advancing the United Nations Sustainable Development Goals, especially Zero Hunger (#2), Good Health and Well-being (#3), Clean Water (#6), Sustainable Cities and Communities (#11), Climate Action (#13), Protecting Life Below Water (#14), Life on Land (#15), and Partnerships for the Goals (#17) (8). By analyzing microbiome interactions across ecosystems, disciplines, and species, One Health enables a holistic understanding of interconnected challenges spanning food security, health, and environmental resilience. Cross-system collaborations enhance the sharing of data, resources, and knowledge, which will accelerate research within and across individual goals.

Here, we highlight microbiome patterns and hypotheses that can be integrated across diverse systems, illustrating the powerful potential of unifying the microbiome sciences. While these exemplars bring testable hypotheses under a One Health framework, they represent a segment of the broader landscape of microbiome-related theories that require a phase of concerted investigation and generalization.

## PHYLOSYMBIOSIS AS A CROSS-SYSTEM TREND

Despite significant advances in microbiome research, many fundamental aspects of the relationship between hosts and their associated microbial communities remain poorly understood. One quantifiable and widely applicable line of inference is phylosymbiosis—a pervasive pattern wherein host phylogeny mirrors microbial community composition (9) (Fig. 1b). Phylosymbiosis incites several experimental and theoretical investigations, such as how does microbial dispersal through ecosystems drive or hinder the onset of phylosymbiosis? Does selection or drift shape phylosymbiosis? Do hosts and microbes dually contribute to shaping phylosymbiotic relationships? From an applied perspective, phylosymbiosis also accentuates the need to consider evolutionary divergence in the efficacy of microbial products across host genetic backgrounds, as tools effective in one system may not be extendable for use in distant relatives.

## STRESS ECOLOGY AND MICROBIOME DYNAMICS

The greatest threat to global health lies in ecological disturbances (e.g., global warming, pollution, and habitat loss), which will inevitably alter microbial eco-evolutionary dynamics and potentially obstruct the flow of microbial diversity across hosts and ecosystems (10). In particular, climate change has far-reaching impacts on microbial communities across the biosphere. Examples include shifts in the cycling of carbon and other essential nutrients, thus resulting in the modulation of ecosystem productivity and greenhouse gas consumption and emission (11). Moreover, climate change can alter the physiology and distribution of pathogenic microbes. For instance, elevated temperatures in North America have extended the seasonal length and survival of ticks (*Ixodes* spp.) that underpin the spread of Lyme disease (*Borrelia burgdorferi*) and anaplasmosis (*Anaplasma phagocytophilum*) (12). Rising temperatures are also predicted to shift the distribution of plant pathogens north, including the destructive oomycete *Phytophthora cinnamomi*, which is included on the “100 of the World’s Worst Invasive Alien Species” list and causes disease in a wide range of trees, shrubs, grasses, and other plants (13). Environmental changes could also cause microbial extinctions, particularly in soil communities (14), and shift host–microbe symbiotic relationships, resulting in devastating consequences, such as coral bleaching (15).

While we foresee challenges in defining categories of “healthy” or “dysbiotic” microbiome states—mostly due to their multiple possible configurations across systems—we can work toward defining compositional and functional norms and cycles, as well as developing frameworks to optimize microbial community resilience with stress and recovery experimentation. This approach will characterize how and why microbiome structural and functional shifts occur, whether these microbiome responses are distinct or consistent across systems, and the consequences for both individual hosts and ecosystem sustainability. For example, a study testing how soil microbial communities collected from 30 grasslands across nine countries respond to simulated extreme environmental stressors discovered highly consistent and phylogenetically conserved microbiome responses (16).

Testing and developing stress ecology theories for microbial systems can help predict how ecosystems respond to perturbations, such as global warming. For instance, the Anna Karenina principle predicts that “healthy” microbial ecosystems have a stable and defined set of variations, while stressed or dysbiotic ecosystems have increased imbalance and variability. This is marked by an increase in beta dispersion and stochasticity, which is commonly observed in disease-impacted plants (17) and animals (18) (Fig. 1c). A potential mechanism driving this observation is the mutualism breakdown hypothesis whereby host–microbe relationships deteriorate under stress (19) (Fig. 1d). A competing idea, the stress gradient hypothesis, argues that the benefits afforded by microbiota are enhanced as host stress levels increase (20) (Fig. 1e). Moreover, evidence suggests that past exposure to stress can influence microbiome trajectories (21) and future microbial community responses to environmental perturbation, a concept known as legacy effects (22) (Fig. 1f). These testable hypotheses provide avenues for developing system-agnostic frameworks to reveal the drivers and impacts of rapid microbial adaptation, which will allow us to predict how different ecosystems will respond based on historical environmental conditions and interactions with global change factors. Projects executed at global levels with standardized approaches and methods are early examples of how the field will more generally deliver the large ambitions of the One Health microbiome sciences, such as the Earth Microbiome Project, Earth Hologenome Initiative, and Global Virome Project (5, 23, 24). By understanding how microbial communities respond to these changes, we can explore their potential for climate change mitigation and restoration.

## CHALLENGES, OPPORTUNITIES, AND A UNIFYING FRAMEWORK

The primary challenge ahead lies in the need to synthesize what we have learned from single-system microbiome studies and boldly launch into a new era that validates theories for multiple microbial systems. The One Health framework, by promoting interconnectedness across systems, offers a conceptual scaffold to overcome these limitations. In acknowledging the current gaps in comparative studies, the intention is not to diminish the elegant single-system studies that are the foundation of the microbiome sciences, but to advocate for the next phase of integrative approaches that recognize the diverse roles of microbes in maintaining healthy states across different scales. This expanded perspective can move towards uncovering the fundamental “rules of life” that govern these complex biological interactions, from organismal immune development to ecosystem resilience—the capacity to maintain or return to a stable state post-disturbance.

Moreover, embracing the One Health perspective emphasizes that Earth’s microbiota are intimately networked, scaling from humans, other animals, and plants to the environment and the biosphere. Indeed, human health cannot be decoupled from the health of other species and ecosystems, as we share microbial lineages, gene pools, and evolutionary histories with the organisms and environments around us. The balance of this fundamentally complex and dynamic biological network of interactions shapes system health, form, and functions. Philosophically, this viewpoint challenges hierarchical perceptions that regard humans as superior (i.e., anthropocentrism) and, in doing so, promotes new paths and possibilities for promoting environmental justice (25). As health disparities rise worldwide, marginalized communities are disproportionately affected by environmental degradation, pollution, and disease. As such, central principles of microbiome science that describe microbial dynamics connecting life forms could have social implications and help drive sustainability efforts. To increase the impact of One Health microbiome research, our efforts must address socioeconomic inequities and sustainability. It is vital for microbiome scientists to work closely with policymakers, clinicians, artists, and general audiences to translate our research into practical solutions.

Unifying microbiome sciences within a One Health framework will require team science to develop and pursue microbiome-focused research questions. Developing interdisciplinary science units and building collaborations at the early stages are critically necessary. In the short term, connecting and comparing systems can be done by repurposing publicly available data. However, inconsistencies in sampling, wet lab, and sequencing methods, as well as a lack of metadata reporting standards, significantly constrain these pursuits. Optimistically, machine learning methods could help reduce the effects of different experimental approaches and data quality, and/or account for inherent cross-system variations. Additionally, a major shift in how interdisciplinary One Health research is funded will be important. Federal funding agencies frequently reinforce research silos with the expectation that a single sector (human, agriculture, or environmental health) will be prioritized. A fully realized One Health research agenda will require a balanced focus that most federal granting systems are not yet widely poised to manage. Private foundations and cross-departmental centers and institutions can help drive these efforts by coordinating and supporting interdisciplinary projects, working groups, and task forces. A notable example is the increasing number of microbiome centers worldwide that are well-positioned to integrate their specialties and resources internationally. There is also momentum at higher levels, such as the National Academies of Sciences, Engineering, and Medicine’s new working group “Exploring Linkages Between Soil Health and Human Health,” and the Netherlands government’s €200 million investment in a One Health Microbiome project called “HOLOmicrobiome Institute.”

Finally, One Health microbiome sciences has the potential to drive a transformative vision with holistic discoveries for predicting health outcomes and developing microbiome-informed health management practices and products. This allows not only microbiologists but also other scientists and practitioners to step away from compartmentalized research into a more holistic and integrative view of the complexity of microbes permeating and modulating the status of the biosphere.
